# *Multidrug resistance-associated protein-1 (MRP1) *genetic variants, MRP1 protein levels and severity of COPD

**DOI:** 10.1186/1465-9921-11-60

**Published:** 2010-05-20

**Authors:** Simona E Budulac, Dirkje S Postma, Pieter S Hiemstra, Lisette IZ Kunz, Mateusz Siedlinski, Henriette A Smit, Judith M Vonk, Bea Rutgers, Wim Timens, H Marike Boezen, the Groningen Leiden Universities Corticosteroids in Obstructive Lung Disease (GLUCOLD) study group

**Affiliations:** 1 Department of Epidemiology, University Medical Center Groningen, University of Groningen, Groningen, the Netherlands; 2 Department of Pulmonology University Medical Center Groningen, University of Groningen, Groningen, the Netherlands; 3 Department of Pulmonology, Leiden University Medical Center, Leiden, the Netherlands; 4 Julius Center for Health Sciences and Primary Care, University Medical Center Utrecht, the Netherlands; 5 Department of Pathology, University Medical Center Groningen, University of Groningen, Groningen, the Netherlands

## Abstract

**Background:**

Multidrug resistance-associated protein-1 (MRP1) protects against oxidative stress and toxic compounds generated by cigarette smoking, which is the main risk factor for chronic obstructive pulmonary disease (COPD). We have previously shown that single nucleotide polymorphisms (SNPs) in *MRP1 *significantly associate with level of FEV_1 _in two independent population based cohorts. The aim of our study was to assess the associations of *MRP1 *SNPs with FEV_1 _level, MRP1 protein levels and inflammatory markers in bronchial biopsies and sputum of COPD patients.

**Methods:**

Five SNPs (rs212093, rs4148382, rs504348, rs4781699, rs35621) in *MRP1 *were genotyped in 110 COPD patients. The effects of *MRP1 *SNPs were analyzed using linear regression models.

**Results:**

One SNP, rs212093 was significantly associated with a higher FEV_1 _level and less airway wall inflammation. Another SNP, rs4148382 was significantly associated with a lower FEV_1 _level, higher number of inflammatory cells in induced sputum and with a higher MRP1 protein level in bronchial biopsies.

**Conclusions:**

This is the first study linking *MRP1 *SNPs with lung function and inflammatory markers in COPD patients, suggesting a role of *MRP1 *SNPs in the severity of COPD in addition to their association with MRP1 protein level in bronchial biopsies.

## Background

Chronic obstructive pulmonary disease (COPD) is an inflammatory lung disease associated with an influx of neutrophils, macrophages and CD8^+ ^T-lymphocytes in the airways and lung tissue[1]. Smoking generates oxidative stress resulting from an oxidant - antioxidant imbalance, and oxidative stress markers are increased in airspaces, blood and urine of smokers and COPD patients[2]. Oxidative stress can be reduced by members of the ATP-binding cassette (ABC) superfamily of transporters. One such a transporter is multidrug resistance-associated protein-1, MRP1, (official name ABCC1, ABC subfamily C, member 1) that plays an important role in normal lung physiology by protecting against toxic xenobiotics and endogenous metabolites[3].

MRP1 was first detected in small cell lung cancer. It has been shown to be highly expressed in the normal human lung [4,5] and particularly at the basolateral side of human bronchial epithelial cells[6]. Interestingly, we have previously shown that MRP1 is less expressed in bronchial epithelium of COPD patients compared to healthy subjects[7]. *Mrp1/Mdr1a/1b *triple knock-out mice had a poor ability for smoke-induced IL-8 production compared with wild type mice, which associated with almost complete absence of inflammatory cells in response to cigarette smoke[8]. An additional study demonstrated that cigarette smoke extract inhibits MRP1 activity in bronchial epithelial cells in vitro[9]. Thus there is a clear role for MRP1 in COPD.

A total of 51 single nucleotide polymorphisms (SNPs) with a minor allele frequency (MAF) > 5% are required to tag the entire *MRP1 *gene in Caucasians[10]. We have shown that two SNPs in *MRP1 *significantly associate with a lower or higher level of FEV_1 _in two independent population-based cohorts. Two additional SNPs had a significant effect of the same, negative magnitude on the level or decline of FEV_1_. One SNP was a significant predictor of COPD in the general population [11].

So far, no study has focused on the relation between *MRP1 *polymorphisms and the level of lung function, inflammatory markers and MRP1 protein in lung tissue of individuals with established COPD. We had the unique opportunity to do so in a recently finished, two center trial in COPD that amongst others studies inflammatory markers in bronchial biopsies and induced sputum[12]. Furthermore, we assessed whether MRP1 protein levels in bronchial biopsies of COPD patients are associated with *MRP1 *SNPs.

## Methods

### Study populations

#### COPD patients

We included 114 patients with COPD who participated in a two-center trial (Groningen Leiden Universities and Corticosteroids in Obstructive Lung Disease; GLUCOLD study). Patient characteristics and methods have been described in detail previously[12]. In brief, all patients had irreversible airflow limitation and chronic respiratory symptoms[13]. Included patients had neither used a course of oral steroids during the previous 3 months, nor maintenance treatment with inhaled or oral steroids during the previous 6 months. They were current or ex-smokers with a smoking history of ≥10 packyears, aged between 45 and 75 years without a history of asthma. The study was approved by the medical ethics committees of the University Medical Centers of Leiden and Groningen. All patients gave their written informed consent.

#### Controls

To verify the differences of MRP1 levels in bronchial biopsies between COPD patients and healthy subjects, we included 37 subjects as controls, of which 28 were previously recruited in order to participate in a smoking cessation program[14]. They were symptomatic and asymptomatic smokers according to the ATS-ERS (American Thoracic Society-European Respiratory Society) guidelines [15] and met the following criteria: 45-75 years of age, >10 pack years of smoking, FEV_1_/FVC pre and post bronchodilator > 70%, no use of inhaled or oral corticosteroids in the previous 6 months, no sign of atopy, no respiratory tract infections one month prior to the study and none of the participants had any co-morbidity[14]. The remaining 9 subjects were included as controls with an FEV_1_/FVC pre and post bronchodilator > 70% and FEV_1 _>80% predicted.

We used an additional control group from the general population-based cohort (Doetinchem) [16] to check for the differences in genotype distributions between COPD patients and general population (Additional file 1).

#### Clinical characteristics

Lung function and reversibility to salbutamol were measured as described previously for COPD patients [12] and for controls[14].

Sputum induction and processing were performed as described previously [12] according to a validated technique[17]. Details on biopsy processing, immunohistology and analysis have been published previously[18]. In brief, we collected the two best morphological biopsies out of four paraffin embedded biopsies per patient and used specific antibodies against T lymphocytes (CD3, CD4 and CD8), macrophages (CD68), neutrophil elastase (NE), mast cell tryptase (AA1) and eosinophils (EG2) (Additional file 1).

#### Selection of the *MRP1 *tagging SNPs and genotyping

We selected SNPs based on our previous results showing a significant association of 5 *MRP1 *SNPs (rs212093, rs4148382, rs35621, rs4781699 and rs504348) with the FEV_1 _level and/or annual FEV_1 _change in two independent population-based cohorts [11]. The rs504348 SNP results in a significant increase in *MRP1 *promoter activity in vitro[19]. Genotyping was performed by K-Bioscience (UK) using their patent-protected competitive allele specific PCR system (KASPar).

#### Biopsies and immunohistochemistry on bronchial biopsies from COPD patients and controls

Details on bronchial biopsy collection and processing are described in the data supplement. Four paraffin-embedded biopsies were cut in 4 μm thick sections and haematoxylin/eosin staining was used for evaluation and selection of the best morphological biopsy per subject for analysis (without crush artefacts, large blood clots, or only epithelial scrapings). The staining was performed on one paraffin section of 4 μm per subject with monoclonal antibody MRPr1 for MRP1 (Santa Cruz, California, USA). Details on immunohistochemical staining are described in Additional file 1.

#### Evaluation of immunohistochemistry on bronchial biopsies from COPD patients and controls

Evaluation of different types of epithelium was performed separately (i.e. basal epithelium, squamous metaplasia, intact epithelium). For the current study, intact bronchial epithelium was selected for analysis.

MRP1 protein was scored for staining intensity in intact epithelium of bronchial biopsies with a semi quantitative score: 0 = no staining; 1 = weak; 2 = moderate; 3 = strong. MRP1 intensity scores for intact epithelium were available from 80 bronchial biopsies of subjects with COPD and 26 bronchial biopsies of controls. Due to the fact that there were only 3 individuals with no immunohistochemical expression of MRP1, the MRP1 intensity was categorised in 3 groups: 1 = weak staining, 2 = moderate staining and 3 = strong staining. Two observers (S.B. and W.T.) performed all evaluations of bronchial biopsies individually, in a blinded manner. Most sections stained variable for MRP1 in epithelium and parts with the most intense staining were evaluated for scoring.

### Statistics

Numbers of inflammatory cells in bronchial biopsies and induced sputum were log transformed to achieve a normal distribution. Linear regression analyses were performed to investigate the association of *MRP1 *SNPs with FEV_1 _level and inflammatory cells (natural logarithm) in bronchial biopsies and induced sputum. Independent variables included in the model were age, gender, height, packyears and genotypes. To assess the effect of SNPs on FEV_1 _level and cell numbers in bronchial biopsies and induced sputum we used the following genetic models:

• General: heterozygote and homozygote variants coded separately as dummy variables and compared to the homozygote wild type

• Dominant: heterozygote and homozygote variants pooled and compared to the homozygote wild type

Differences in MRP1 staining intensity between biopsies of COPD patients and controls and according to *MRP1 *SNPs were analyzed using Chi-square tests. Analyses were performed using SPSS version 16.0 for Windows and values of p < 0.05 (tested 2-sided) were considered statistically significant.

## Results

The clinical characteristics of COPD patients and controls are presented in Table 1.

**Table 1:** Clinical characteristics of COPD patients and controls with airway biopsy available.

	COPD patients (n = 114)	Controls (n = 37)
Males, n (%)	99 (86.8)	16 (43.2)

Age (years)	61.6 ± 7.7	52.3 ± 5.5

Height (cm)	175.5 ± 7.8	172.8 ± 10

Packyears¶	41.8 (31.2 - 54.7)	25.4 (20.2-35.0)

Current smoker, n (%)	72 (63.2)	30 (81.1)

FEV_1_/FVC (%)	49.5 ± 8.8	77.2 ± 6.1

FEV_1 _(L)	1.8 ± 0.4	3.2 ± 0.8

FEV_1 _% pred.*	56 ± 10	100 ± 14

MRP1 level ^#^, n	80	26

Data are presented as mean ± standard deviation or ¶ median (25^th ^- 75^th ^percentile); FEV_1 _= forced expiratory volume in one second; FEV_1_/FVC = FEV_1_/forced vital capacity; * % pred. = percentage of predicted value; ^# ^refers to the number of individuals having bronchial biopsies with available MRP1 levels of intensity; MRP1 = multidrug resistance-associated protein-1

DNA was available from 110 out of 114 COPD patients and from 37 controls. All 5 *MRP1 *SNPs were in Hardy Weinberg Equilibrium (HWE, p > 0.05) and were not highly correlated with each other (the highest r^2 ^in our population is 0.34) (See figure S1 in Additional file 2). There were no significant differences in genotype distributions between the COPD patients and the general population-based control cohort (Additional file 1). Likewise, there were no significant differences in genotype distributions between the COPD patients and controls (Table 2).

**Table 2:** Prevalence of *MRP1 *SNPs in COPD patients and controls.

		COPD patients n = 110 (%)	Controls n = 37 (%)	p value
rs212093	AA	37 (33.9)	8 (25.0)	0.55
	
	AG	50 (45.9)	18 (56.2)	
	
	GG	22 (20,2)	6 (18.8)	

rs4148382	GG	83 (76.1)	29 (82.8)	0.12
	
	GA	26 (23.9)	5 (14.3)	
	
	AA	-	1 (2.9)	

rs504348	CC	78 (72.2)	22 (71.0)	0.23
	
	CG	27 (25.0)	6 (19.3)	
	
	GG	3 (2.8)	3 (9.7)	

rs4781699	GG	58 (52.7)	21 (61.8)	0.47
	
	GT	45 (40.9)	10 (29.4)	
	
	TT	7 (6.4)	3 (8.8)	

rs35621	CC	89 (80.9)	30 (85.7)	0.74
	
	CT	20 (18.2)	5 (14.3)	
	
	TT	1 (0.9)	-	

Different numbers for the SNP genotypes are due to missing genotype data

SNP = single nucleotide polymorphism; *MRP1 *= *multidrug resistance-associated protein-1*

Table 3 shows the number and the percentage of inflammatory cells in bronchial biopsies and induced sputum from the COPD patients.

**Table 3:** The number of inflammatory cells in bronchial biopsies and induced sputum of COPD patients

Bronchial biopsies	Absolute numbers per 0.1 mm^2 ^sub-epithelial area	
	
CD3	123.5 (69.2 - 182.5)	
	
CD4	48.0 (27.7 - 72.0)	
	
CD8	21.5 (11.0 - 37.2)	
	
Plasma cells	8.5 (3.5 - 14.5)	
	
Mast cells	26.5 (19.0 - 34.5)	
	
Macrophages	8.5 (4.5 - 13.0)	
	
Neutrophils	4.0 (2.0 - 8.4)	
	
Eosinophils	1.5 (0.5 - 4.2)	
Induced sputum	Absolute numbers (10^4^/ml)	Percentage (%)

Total cell count*	139.7 (77.9 - 311.3)	

Neutrophils	101.6 (46.8 - 228.5)	72.8 (59.9 - 81.7)

Macrophages	31.1 (17.9 - 61.1)	22.1 (14.8 - 33.2)

Eosinophils	1.3 (0.4 - 4.5)	1.1 (0.3 - 2.2)

Lymphocytes	2.2 (1.1 - 6.8)	1.7 (1.2 - 2.3)

Epithelial cells	1.4 (0.6 - 3.4)	1.0 (0.3 - 2.3)

Data are presented as median (25^th ^- 75^th ^percentile)

*Total cell count refers to the number of non-squamous cells in induced sputum

### *MRP1 *SNPs and FEV_1 _level in COPD patients

**Figure 1:**
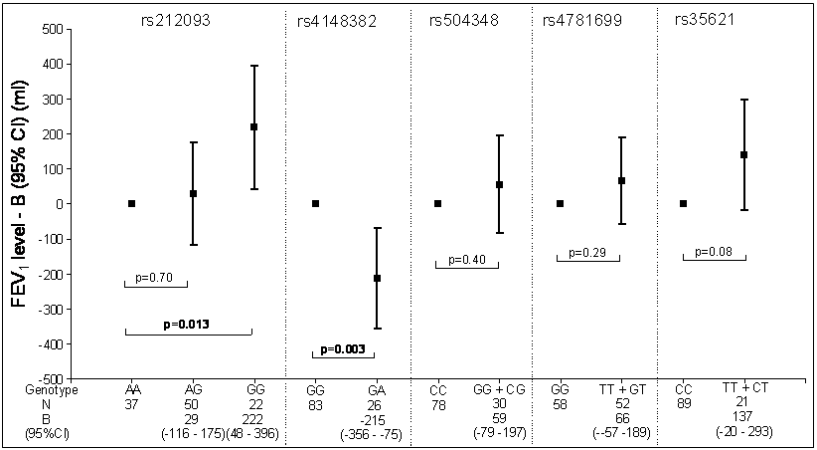
**Estimated effects of *MRP1 *genotypes on level of FEV_1 _in COPD patients**. FEV_1 _= forced expiratory volume in one second. N= Number of individuals. Squares represent the regression coefficient (B) and vertical bars represent 95% confidence interval (CI). Wild type was set to zero as the reference category. The analyses are adjusted for age, gender, height and packyears.

In a general model, individuals who were homozygote mutant (GG) for rs212093 had a significantly higher FEV_1 _than wild type (AA) individuals, as reflected by a regression coefficient B value (95% CI, confidence interval) of 222 ml (48 ml to 396 ml); p = 0.013. Heterozygote (GA) individuals for rs4148382 had a significantly lower FEV_1 _than wild type (GG) individuals (-215 ml (-356 ml to -75 ml); p = 0.003). None of the other 3 SNPs (rs504348, rs4781699 and rs35621) was significantly associated with the FEV_1 _level (Figure 1). Additional adjustment for current smoking status did not change the size or significance of the effect estimates of the genotypes on level of FEV_1_.

### *MRP1 *SNPs and inflammatory cells in bronchial biopsies in COPD patients

**Figure 2:**
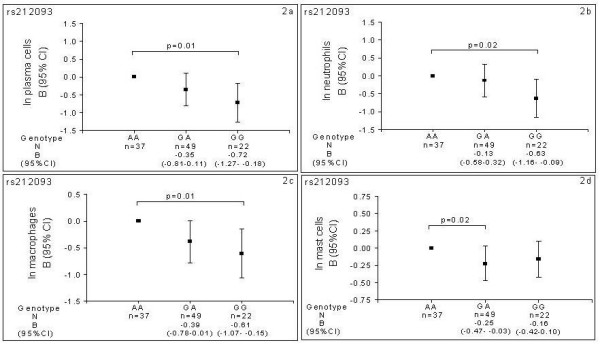
**Estimated effects of *MRP1 *genotypes on inflammatory cells in bronchial biopsies of COPD patients**. 2a: Number of plasma cells according to rs212093 genotype. 2b: Number of neutrophils according to rs212093 genotype. 2c: Number of mast cells according to rs212093 genotype. 2d: Number of macrophages according to for rs212093 genotype.

Homozygote mutant (GG) individuals for rs212093 had a significantly lower number of plasma cells (-0.72 (-1.27 to -0.18); p = 0.01), neutrophils (-0.63 (-1.16 to -0.09); p = 0.02) and macrophages (-0.61(-1.07 to -0.15); p = 0.01) in bronchial biopsies than wild type (AA) individuals (Figures 2a, b and 2c, respectively). Individuals who were heterozygote (AG) for rs212093 had lower numbers of mast cells than wild type (AA) individuals (-0.25 (-0.47 to -0.03); p = 0.02) (Figure 2d).

**Figure 3:**
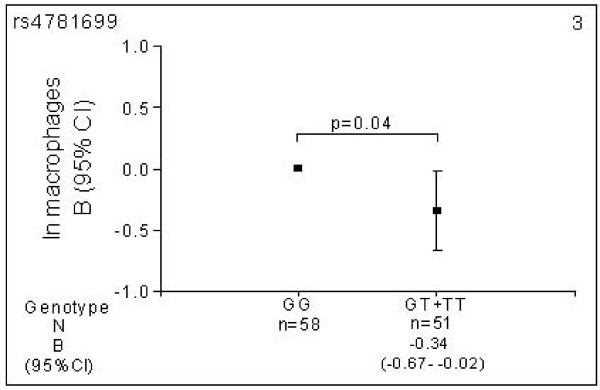
**Estimated effects of *MRP1 *genotypes on inflammatory cells in bronchial biopsies of COPD patients**. Number of macrophages according to rs4781699 genotype. N = number of individuals. Data are presented as natural logarithm of each type of cells in bronchial biopsies. Different numbers for the SNP genotypes are due to missing data on genotype or inflammatory cells. Squares represent the regression coefficient (B) and vertical bars the 95% confidence interval (CI). Wild type was set to zero as the reference category. The analyses are adjusted for age, gender and packyears.

Minor allele carriers (GT/TT) for rs4781699 had significantly lower numbers of macrophages (-0.34 (-0.67 to -0.02); p = 0.04) than wild type (GG) individuals (Figure 3).

The genotypes for the other two SNPs (rs4148382 and rs35621) were not significantly associated with any of the inflammatory cells in the bronchial biopsies.

### *MRP1 *SNPs and inflammatory cells in sputum in COPD patients

Heterozygote (GA) individuals for rs4148382 had a significantly higher total cell count (0.59 (0.11 to 1.07) p = 0.01) and neutrophils (0.61 (0.06 to 1.16); p = 0.03) in sputum compared to wild type (GG) individuals. None of the other SNPs was significantly associated with inflammatory cells in sputum.

Additional adjustment for current smoking status did not change the size or significance of the effect estimates of the genotypes on inflammatory cells in bronchial biopsies and in induced sputum.

Detailed data on the *MRP1 *genotypes and inflammatory cells in bronchial biopsies and induced sputum are presented in the Additional file 1.

### MRP1 protein levels in COPD patients and controls

There were no significant differences in MRP1 protein levels between COPD patients and controls.

**Figure 4:**
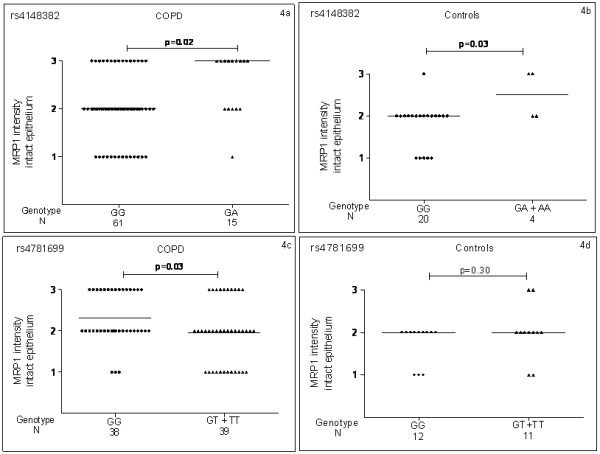
***MRP1 *SNPs and MRP1 protein levels of COPD patients and controls**. 4a: MRP1 protein levels according to rs4148382 genotype in COPD patients. 4b: MRP1 protein levels according to rs4148382 genotype in controls. 4c: MRP1 protein levels according to rs4781699 genotype in COPD patients. 4d: MRP1 protein levels according to rs4781699 genotype in controls. N= number of individuals.

Heterozygote (GA) individuals for rs4148382 had a significantly higher MRP1 protein level than wild type (GG) individuals in COPD patients (p = 0.026) (Figure 4a) and in the control group minor allele carriers (GA/AA) for rs4148382 had a significantly higher MRP1 protein level than wild type (GG) individuals (p = 0.037) (Figure 4b).

Minor allele carriers (GT/TT) for rs4781699 had significantly lower MRP1 protein level than wild type (GG) individuals in COPD patients (p = 0.036) (Figure 4c), but there was no significant difference in MRP1 protein level in the control group (Figure 4d).

None of the other 3 SNPs (rs212093, rs504348 and rs35621) associated significantly with MRP1 protein levels. Levels of MRP1 were not related to lung function parameters, inflammatory cells in bronchial biopsies or number of packyears.

## Discussion

**Figure 5:**
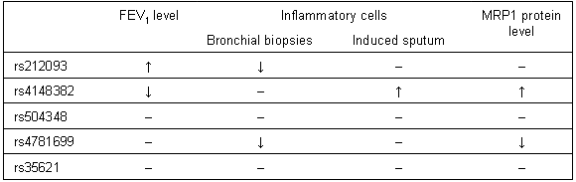
**Summary of *MRP1 *SNPs' associations for COPD patients**. FEV_1 _= forced expiratory volume in one second; MRP1 = multidrug resistance-associated protein-1; ↑ = positive association; ↓ = negative association; - = no association.

This is the first study linking *MRP1 *SNPs with the severity of COPD and additionally with the intensity of MRP1 staining in bronchial biopsies. Our results suggest a role of *MRP1 *in COPD severity, as indicated by the associations of rs212093 genotypes with a higher level of FEV_1 _and less inflammatory cells in bronchial biopsies. Additionally, the SNPs rs504348 and rs4781699 were associated with less airway wall inflammation and rs4148382 with a lower FEV_1 _level and increased sputum cell numbers. Moreover, the before mentioned SNPs rs4148382 and rs4781699 were associated with respectively higher and lower levels of MRP1 protein in bronchial biopsies of COPD patients (see summary of the results in Figure 5).

Since first described in 1992 [4], a fair amount of data on the structure, substrate, function, and regulation of this transporter has been gathered. MRP1 is a member of the human ATP-binding cassette superfamily of transporters which regulates the traffic of molecules across cell membranes. The MRP1 pump confers resistance to several chemotherapeutic agents including vincristine, daunorubicin and methotrexate[20,21]. In addition to protecting cells within the body against drugs, environmental toxins and heavy metals, MRP1 has been suggested to be involved in the cellular oxidative defence system and inflammation [22,23], both being important in COPD development and progression.

We showed that the *MRP1 *polymorphism rs212093 was significantly associated with a higher FEV_1 _level. In line with this, rs212093 SNP was associated with lower numbers of plasma cells, macrophages, neutrophils and mast cells in bronchial biopsies, cells implicated in COPD previously. Increased numbers of neutrophils have been reported in bronchial biopsies of smokers with airflow limitation, an increase that was associated with a lower FEV_1_[24]. Neutrophils and macrophages release proteolytic enzymes and generate oxidants, which cause tissue damage as well as cytokines and chemokines that can potentiate inflammation and trigger an immune response. We previously reported a larger number of B lymphocytes in bronchial biopsies of patients with COPD than in controls without airflow limitation[25]. Furthermore, epithelial cells of smokers with COPD contain higher macrophage and mast cell numbers than smokers without COPD[26]. In a triple knock-out mouse model, we previously demonstrated that the inflammatory response to inhalation of cigarette smoke is reduced when MRP1 is absent[8]. Linking previously reported increased airway wall inflammation in COPD with genetic variants of *MRP1 *we found rs212093 to be associated with lower numbers of inflammatory cells in bronchial biopsies, therefore, this SNP might play a protective role in COPD. This SNP located in 3'region is known to be in complete linkage disequilibrium with rs129081 located in the 3' untranslated region [10] and therefore this polymorphism might be involved in the regulation of MRP1 mRNA stability[11].

One could raise the issue of multiple testing and that we should have adjusted for this in our analyses, but we feel that applying a sequential (classical) Bonferroni correction is not appropriate in the current dataset for a number of reasons[27]. Firstly, our choice for the current study was explicitly driven by our previous findings, suggesting that there might be associations between *MRP1 *SNPs and COPD severity. Thus, we explicitly hypothesized on the main outcome variables on forehand. Secondly, a Bonferroni correction would not take into account the potential clustering of outcome variables, which might occur jointly at high or low levels, e.g. a Pearson's correlation coefficient r = 0.79 for macrophages and lymphocytes in induced sputum, or are defined as each others ratios[27]. This suggests one might preferentially test a cluster of outcome variables as "one outcome variable" rather than test all variables separately.

It has been shown previously that higher neutrophil percentages in induced sputum correlate with lower FEV_1 _levels [28], therefore it is of interest that rs4148382, located in 3'region of *MRP1*, is associated significantly with higher total cells counts and neutrophils in induced sputum and lower FEV_1 _level. The association with total cell counts might be driven by the neutrophils which represent 72% of the total cells in induced sputum. The functional consequence of this particular SNP is not known so far and it is not known whether any functional polymorphism is in linkage disequilibrium with it. This polymorphism is located closely to the 5'end of the *MRP6 *which maps also on chromosome 16. However, MRP6 mRNA is moderately present in human lung extracts [29] and highly expressed in the liver and kidney [6], which might suggest indeed that the effect of this particular SNP is within *MRP1 *and not *MRP6*. How this SNP functionally contributes to COPD severity has to be further unravelled in future studies.

The observed effects in the current study appear to be opposite to previous findings in the same general population as described by Siedlinski et al[11]. In the current study, which extends the previous findings, we observed that rs4148382 associated with a lower FEV_1 _level in COPD patients, whereas in the general population from the Doetinchem study rs4148382 associated with a higher FEV_1 _level [11]. With respect to these findings it is worth mentioning that the present study was not designed to compare the direction or magnitude of effect estimates between the COPD patients and general population with respect to FEV_1 _and genetic factors. The opposite effects are likely due to the fact that we selected a COPD subset of the Doetinchem general population for the current study by matching on the clinical characteristics age, number of packyears and FEV_1_/FVC<70%. Although both groups had almost the same number of packyears (median 25^th ^- 75^th ^percentile) (40 (34.1 - 48.7) vs. 41.8 (31.2 -54.7)), the matched COPD subset in the general population had a higher lung function (mean FEV_1 _% predicted (SD) = 79.7 (13.4)) than our current COPD patients (49.5 (8.8)). This suggests that the COPD subset of subjects from the Doetinchem study who, fulfilled the GOLD criteria of COPD, might be less susceptible to cigarette smoke and COPD development. Therefore, the patients included in the current study with established COPD were probably not comparable with the heavy smokers from the general population based control cohort (Doetinchem).

Additionally, we have calculated the haplotypes of *MRP1 *and assessed the effects of these haplotypes on FEV_1 _level and inflammatory cells in bronchial biopsies and induced sputum. We observed that the effects of *MRP1 *haplotypes are due to the specific SNP constituting these haplotypes, and therefore didn't add new information. Details on the *MRP1 *haplotypes are presented in the Additional file 1.

Decreased or increased functional MRP1 expression may have a high impact on development and/or progression of lung diseases and protection against air pollution and inhaled toxic compounds such as present in cigarette smoke[6,7,30]. One of our earlier studies showed that the MRP1 intensity in bronchial biopsies of COPD patients was lower compared to healthy individuals[7]. How can we reconcile this with our current findings of MRP1 staining in COPD patients and controls? One option is that this might be due to differences in staining between paraffin and frozen biopsies[31]. More important, it might be due to underlying differences of *MRP1 *genotyping distribution in the two populations. It appeared that the previous low intensity of MRP1 staining was driven by wild type individuals [7] and if we would have known this at that time, it might have had a different impact on the interpretation of the results. MRP1 is an essential pump for glutathione (GSH) - conjugates such as the inflammatory mediator leukotriene C4 (LTC4) as well as substrates in the presence of GSH (i.e. glutathione disulphide, GSSG) [32], thereby decreasing intracellular concentrations of toxic compounds. Given the rarity of homozygote mutant (AA) individuals for rs4148382 all the conclusions about this SNP are drawn based on the heterozygote (GA) individuals in COPD patients. It might be that in particular individuals who are heterozygote for rs4148382 SNP can have a locally high MRP1 protein level which therefore might lead to more severe inflammation at that site. Clearly, further research needs to investigate this approach in a larger sample of subjects with or without COPD.

## Conclusions

In conclusion, our study is the first to demonstrate that *MRP1 *plays a role in COPD severity, given the association of polymorphisms in *MRP1 *with airway wall inflammation, the level of lung function and moreover MRP1 protein levels in subjects with established COPD. This is an important step forward linking *MRP1 *polymorphisms with the pathophysiology of COPD.

## Competing interests

The authors declare that they have no competing interests.

## Authors' contributions

SEB wrote the manuscript. SEB, JMV, and HMB analyzed the data. DSP, PSH, JMV, HMB designed the GLUCOLD cohort study. JMV managed the data. HAS designed the Doetinchem cohort study and managed the data. SEB, DSP, MS, JMV, WT, HMB interpreted the data. SEB and BR performed immunohistochemistry. SEB and WT interpreted the results of the immunohistochemistry. All authors proposed corrections and approved the final version of the manuscript.

## Supplementary Material

Additional file 1 ***MRP1*****genetic variants, MRP1 protein levels and severity of COPD.** Click here for file

Additional file 2 Figure S1: Linkage disequilibrium plot and correlation coefficients (r^2^) for 5 *MRP1 *polymorphisms genotyped in COPD patients (n = 110). Click here for file
