# Supplementary material for: Multidrug resistance-associated protein-1 (MRP1) genetic variants, MRP1 protein levels and severity of COPD
Source: Respir Res. 2010 May 20;11(1):60. doi: 10.1186/1465-9921-11-60 (PMC2882908; doi:10.1186/1465-9921-11-60)
Supplement: Additional file 1 — MRP1genetic variants, MRP1 protein levels and severity of COPD. [file 1465-9921-11-60-S1.DOC]

**Data supplement:**

***Multidrug resistance-associated protein-1 (MRP1)* genetic variants, MRP1 protein levels and severity of COPD**

Simona E Budulac^1^, Dirkje S Postma^2^, **Pieter S Hiemstra^3^, Lisette IZ Kunz^3^, Mateusz Siedlinski^1^, Henriette A Smit^4^**, Judith M Vonk^1^, Bea Rutgers^5^, Wim Timens^5^**, H Marike** Boezen^1§^ and the GLUCOLD study group^6^

^1^Department of Epidemiology, University Medical Center Groningen, University of Groningen, Groningen, The Netherlands

^2^Department of Pulmonology University Medical Center Groningen, University of Groningen, Groningen, The Netherlands

^3^Department of Pulmonology, Leiden University Medical Center, Leiden, The Netherlands

^4^Julius Center for Health Sciences and Primary Care, University Medical Center Utrecht, The Netherlands

^5^Department of Pathology, University Medical Center Groningen, University of Groningen, Groningen, The Netherlands

^6^The GLUCOLD study group: Groningen Leiden Universities Corticosteroids in Obstructive Lung Disease

^§^Corresponding author

Email addresses:

SEB: [s.budulac@epi.umcg.nl](mailto:s.budulac@epi.umcg.nl)

DSP: [d.s.postma@int.umcg.nl](mailto:d.s.postma@int.umcg.nl)

PSH: [P.S.Hiemstra@lumc.nl](mailto:P.S.Hiemstra@lumc.nl)

LIZK: [L.I.Z.Kunz@lumc.nl](mailto:L.I.Z.Kunz@lumc.nl)

MS: [m.siedlinski@epi.umcg.nl](mailto:m.siedlinski@epi.umcg.nl)

HAS: [h.a.smit@umcutrecht.nl](mailto:h.a.smit@umcutrecht.nl)

JMV: [j.m.vonk@epi.umcg.nl](mailto:j.m.vonk@epi.umcg.nl)

BR: [b.rutgers@path.umcg.nl](mailto:b.rutgers@path.umcg.nl)

WT: [w.timens@path.umcg.nl](mailto:w.timens@path.umcg.nl)

HMB: [h.m.boezen@epi.umcg.nl](mailto:h.m.boezen@epi.umcg.nl)

**METHODS**

**Population-based control cohort (Doetinchem)**

A population based control group was included in the current study to check for differences in genotype distribution between COPD patients and the general population based. We selected 262 subjects as controls from this general population-based control Doetinchem cohort [1] that were comparable with the COPD patients with regard to their clinical characteristics (age between 45 and 75 years, smoking >10 pack years, FEV_1_/FVC>70%).

**Bronchoscopy and biopsy processing of COPD patients and control group**

Fiberoptic bronchoscopy, biopsy processing, immunohistology and analysis were performed as described previously. [2] In brief, as previously described, paraffin embedded biopsies were cut in 4μm thick sections and haematoxylin/eosin staining was used for evaluation and selection of the two morphological best biopsies per patient for analysis (without crushing artefacts, large blood clots, or only epithelial scrapings). [2] Specific antibodies were used against T lymphocytes (CD3 (DAKO, Glostrup, Denmark), CD4 (Novocastra, UK) and CD8 (DAKO, Glostrup, Denmark)), macrophages (CD68 (DAKO, Glostrup, Denmark)), neutrophil elastase (NE (DAKO, Glostrup, Denmark)), mast cell tryptase (AA1 (DAKO, Glostrup, Denmark)) and eosinophils (EG2 (Pharmacia Diagnostics, Uppsala, Sweden)). [2]

Bronchoscopy, biopsy collection and processing were performed for the controls in a similar way as for the COPD group and were previously described in detail.[3]

**Immunohistochemistry on bronchial biopsies from COPD patients and controls**

The immunohistochemical staining was performed on one paraffin section of 4μm per subject with monoclonal antibody MRPr1 for MRP1 purchased from Santa Cruz Biotechnology, Inc. Initially, the tissue slides were deparaffinised with xylene (10 min.) and rehydrated in graded alcohols before staining. Slides were pre-treated with 1mM EDTA buffer in the microwave for 15 minutes. The staining was performed automatically using the Dako Autostainer (DAKO, Copenhagen, Denmark). Pre-incubation with peroxidise blocking reagent (DAKO, Copenhagen, Denmark) was used for blocking endogenous peroxidase. First antibody used was monoclonal antibody MRPr1 for MRP1 with a dilution of 1:50, the second antibody used was polyclonal antibody GaR^po^ (goat anti-rabbit peroxidase, DAKO, Copenhagen, Denmark) with a dilution of 1:100 and the third antibody used was polyclonal antibody RaG^po^ (rabbit anti-goat peroxidase, DAKO, Copenhagen, Denmark) with a dilution of 1:100. Nova Red (SK4800, Vector, USA) was used as a substrate giving a reddish-brown reaction product. Counterstaining was performed using Mayer’s haematoxylin. A negative control was obtained by omission of the primary antibody.

**Statistics**

Differences in genotypes distribution between COPD patients and population-based controls were tested using Chi-square tests.

***MRP1* haplotypes for COPD patients**

We calculated the haplotypes of *MRP1* and additionally, we investigated the effects of each of the *MRP1* haplotypes on FEV_1_ level, inflammatory cells in bronchial biopsies and induced sputum. The most frequent haplotype e.g. 00000, was set as a reference (‘wild type haplotype’). For these analyses we used HAPSTAT version 3.0.

**RESULTS**

5 *MRP1* SNPs were in Hardy Weinberg Equilibrium (HWE, p>0.05) and were not highly correlated with each other (r^2^ <0.8). (Figure S1)

The clinical characteristics of COPD patients and general population-based controls (Doetinchem) are presented in table S1.

There were no significant differences in genotype distributions between COPD patients and the general population based control group. (Table S2)

The association of *MRP1* SNPs and FEV_1_ level, inflammatory cells in the bronchial biopsies and in induced sputum in subjects with established COPD are presented in the tables S3a, S3b respectively S4a, S4b and S5.

We determined the haplotypes of *MRP1* and table S6 shows the frequencies of *MRP1* haplotypes for each of the outcomes e.g. FEV_1_ level, inflammatory cells in bronchial biopsies and induced sputum. Compared to the wild type haplotype (00000), none of the other *MRP1* haplotypes had a significant effect on FEV_1_ level. The observed effects of *MRP1* haplotypes were in line with our main findings on the effects of *MRP1* SNPs on inflammatory cells in bronchial biopsies and induced sputum: The 01000 haplotype was significantly associated with a higher number of total cells in induced sputum (B (95%CI) =0.59 (0.08 -1.10); p=0.02) as compared to the 00000 haplotype.

**Tables**

Table S1: Clinical characteristics of COPD patients and general population

|  | COPD patients  (n= 114) | General population  (n=262) |
| --- | --- | --- |
| Males, n (%) | 99 (86.8) | 147 (56.1) |
| Age (years) | 61.6 ± 7.7 | 54.9 ± 6.7 |
| Height (cm) | 175.5 ± 7.8 | 173.3 ± 8.5 |
| Pack-years¶ | 41.8 (31.2 – 54.7) | 23.5 (15.9 – 32.0) |
| Current smoker, n (%) | 72 (63.2) | 115 (43.9) |
| FEV_1_/FVC (%) | 49.5 ± 8.8 | 78.6 ± 4.8 |
| FEV_1_ (L) | 1.8 ± 0.4 | 3.3 ± 0.7 |
| FEV_1_ % pred.* | 56 ± 10 | 105 ± 14 |

FEV_1_ = forced expiratory volume in one second

FEV_1_/FVC = forced expiratory volume in one second/forced vital capacity

* % pred. = percentage of predicted value.

**Table S2: Prevalence of *MRP1* SNPs in COPD patients and general population**

| SNP |  | COPD patients  n=110 (%) | General population  n=262 (%) | p value |
| --- | --- | --- | --- | --- |
| rs212093 | AA | 37 (33.9) | 81 (31.4) | 0.85 |
|  | GA | 50 (45.9) | 126 (48.8) |
|  | GG | 22 (20.2) | 51 (19.8) |
| rs4148382 | GG | 83 (76.1) | 213 (82.2) | 0.21 |
|  | GA | 26 (23.9) | 44 (17.0) |
|  | AA | - | 2 (0.8) |
| rs504348 | CC | 78 (72.2) | 167 (64.2) | 0.32 |
|  | CG | 27 (25.0) | 82 (31.6) |
|  | GG | 3 (2.8) | 11 (4.2) |
| rs4781699 | GG | 58 (52.7) | 128 (49.6) | 0.51 |
|  | GT | 45 (40.9) | 104 (40.3) |
|  | TT | 7 (6.4) | 26 (10.1) |
| rs35621 | CC | 89 (80.9) | 203 (79.3) | 0.92 |
|  | CT | 20 (18.2) | 50 (19.5) |
|  | TT | 1 (0.9) | 3 (1.2) |

SNP = single nucleotide polymorphism

*MRP1* = multidrug resistance-associated protein-1

**Table S3a: *MRP1* SNPs and inflammatory cells in bronchial biopsies of COPD patients**

| **SNP** | **Model** | **Ln(CD3^+)^** | **Ln(CD4^+^)** | **Ln(CD8^+^)** | **Ln(plasma cells)** |
| --- | --- | --- | --- | --- | --- |
| rs212093 | general model a | -0.11 (-0.39-0.17) | -0.14 (-0.48-0.20) | -0.19 (-0.84-0.46) | -0.35 (-0.81-0.11) |
|  | general model b | -0.27 (-0.61-0.06) | -0.36 (-0.77-0.05) | -0.38 (-1.14-0.38) | **-0.72 (-1.27- -0.18)** |
|  | dominant | -0.17 (-0.43-0.09) | -0.22 (-0.53-0.10) | -0.26 (-0.89-0.34) | **-0.48 (-0.91- -0.05)** |
| rs4148382 | dominant | 0.16 (-0.13-0.45) | 0.27 (-0.07-0.62) | 0.43 (-0.22-1.07) | 0.07 (-0.41-0.54) |
| rs504348 | general model a | -0.03 (-0.31-0.25) | 0.06 (-0.27-0.39) | 0.07 (-0.57-0.70) | -0.03 (-0.31-0.25) |
|  | general model b | -0.46 (-1.20-0.28) | **-0.98 (-1.87- -0.09)** | -0.30 (-1.98-1.39) | -0.46 (-1.20-0.28) |
|  | dominant | -0.07 (-0.34-0.20) | -0.04 (-0.36-0.29) | 0.03 (-0.58-0.64) | -0.14 (-0.58-0.30) |
| rs4781699 | general model a | -0.13 (-0.38-0.12) | -0.17 (-0.47-0.13) | -0.45 (-1.01-0.11) | -0.23 (-0.65-0.18) |
|  | general model b | -0.15 (-0.65-0.35) | -0.27 (-0.88-0.33) | -0.01 (-1.12-0.12) | 0.08 (-0.76-0.91) |
|  | dominant | -0.13 (-0.37-0.11) | -0.18 (-0.47-0.10) | -0.39 (-0.92-0.15) | -0.19 (-0.59-0.21) |
| rs35621 | general model a | -0.14 (-0.45-0.17) | 0.01(-0.37-0.38) | -0.54 (-1.24-0.15) | -0.18 (-0.69-0.34) |
|  | general model b | -0.14 (-1.40-1.13) | 0.06 (-1.48-1.59) | 1.18 (-1.65-0.02) | 1.19 (-0.92-3.29) |
|  | dominant | -0.14 (-0.44-0.17) | 0.01 (-0.37-0.38) | -0.47 (-1.16-0.22) | -0.13 (-0.64-0.38) |

Data are presented as B (95%CI) = regression coefficient (95% confidence interval); ln=natural logarithm;

The analyses are adjusted for age, gender and packyears

General model a: heterozygote versus wild type

General model b: homozygote mutant versus wild type

SNPs showing a significant effect are depicted in bold (p<0.05)

**Table S3b: *MRP1* SNPs and inflammatory cells in bronchial biopsies of COPD patients**

| **SNP** | **Model** | **Ln(mast cells)** | **Ln(macrophages)** | **Ln(neutrophils)** | **Ln(eosinophils)** |
| --- | --- | --- | --- | --- | --- |
| rs212093 | general model a | **-0.25 (-0.47- -0.03)** | -0.39 (-0.78-0.01) | -0.13 (-0.58-0.32) | 0.45 (-0.65-1.56) |
|  | general model b | -0.16 (-0.42-0.10) | **-0.61 (-1.07- -0.15)** | **-0.63 (-1.16- -0.09)** | -0.20 (-1.50-1.10) |
|  | dominant | **-0.22 (-0.42- -0.02)** | **-0.47 (-0.83- -0.11)** | -0.30 (-0.72-0.12) | 0.23 (-0.78-1.24) |
| rs4148382 | dominant | -0.01 (-0.23-0.22) | 0.23 (-0.18-0.63) | 0.12 (-0.35-0.58) | 0.43 (-0.66-1.51) |
| rs504348 | general model a | 0.06 (-0.15-0.28) | 0.03 (-0.36-0.42) | -0.01 (-0.46-0.44) | **-1.09 (-2.15- -0.04)** |
|  | general model b | 0.25 (-0.33-0.84) | -0.18 (-1.23-0.86) | -0.64 (-1.83-0.56) | 0.59 (-2.23-3.41) |
|  | dominant | 0.08 (-0.13-0.29) | 0.01 (-0.37-0.38) | -0.07 (-0.50-0.37) | -0.94 (-1.96-0.09) |
| rs4781699 | general model a | -0.02 (-0.21-0.18) | **-0.39 (-0.73- -0.05)** | -0.13 (-0.53-0.27) | -0.45 (-1.41-0.51) |
|  | general model b | 0.09 (-0.31-0.48) | -0.06 (-0.74-0.63) | -0.18 (-0.98-0.63) | -0.34 (-2.26-1.59) |
|  | dominant | -0.01 (-0.19-0.18) | **-0.34 (-0.67- -0.02)** | -0.14 (-0.52-0.25) | -0.43 (-1.35-0.48) |
| rs35621 | general model a | 0.16 (-0.09-0.40) | -0.10 (-0.53-0.33) | -0.38 (-1.58-0.82) | -0.38 (-1.58-0.82) |
|  | general model b | 0.33 (-0.66-1.32) | 1.48 (-0.28-3.24) | 0.78 (-4.10-5.65) | ,778 (-4.10-5.65) |
|  | dominant | 0.16 (-0.07-0.40) | -0.04 (-0.47-0.39) | -0.33 (-1.51-0.85) | -0.33 (-1.51-0.85) |

Data are presented as B (95%CI) = regression coefficient (95% confidence interval); ln=natural logarithm

The analyses are adjusted for age, gender and packyears

General model a: heterozygote versus wild type

General model b: homozygote mutant versus wild type

SNPs showing a significant effect are depicted in bold (p<0.05)

**Table S4a: *MRP1* SNPs and inflammatory cells in induced sputum of COPD patients**

| **SNP** | **Model** | **Ln (total cells count¶)** | **Ln (neutrophils)** | **Ln (macrophages)** | **Ln (eosinophils)** |
| --- | --- | --- | --- | --- | --- |
| rs212093 | General model a | -0.09 (-0.58-0.41) | -0.17 (-0.73-0.39) | 0.06 (-0.44-0.55) | 0.08 (-0.95-1.12) |
|  | General model b | -0.21 (-0.79-0.37) | -0.23 (-0.89-0.43) | -0.20 (-0.78-0.37) | 0.02 (-1.18-1.22) |
|  | dominant | -0.13 (-0.59-0.33) | -0.19 (-0.71-0.33) | -0.03 (-0.49-0.42) | 0.06 (-0.88-1.01) |
| rs4148382 | dominant | **0.59 (0.11-1.07)** | **0.61 (0.06-1.16)** | 0.46 (-0.02-0.94) | 0.41 (-0.62-1.43) |
| rs504348 | General model a | 0.03 (-0.45-0.50) | -0.01 (-0.55-0.53) | 0.12 (-0.35-0.59) | 0.65 (-0.34-1.63) |
|  | General model b | -1.04 (-2.51-0.44) | -1.15 (-2.82- 0.52) | -1.15 (-2.61-0.31) | 0.41 (-2.66-3.47) |
|  | dominant | -0.05 (-0.51-0.41) | -0.09 (-0.62-0.43) | 0.03 (-0.43-0.49) | 0.63 (-0.33-1.59) |
| rs4781699 | General model a | -0.03 (-0.45-0.38) | 0.01 (-0.46-0.48) | -0.08(-0.49-0.32) | 0.81 (-0.03-1.66) |
|  | General model b | -0.74 (-1.79-0.31) | 0.01 (-1.88-0.51) | -0.10(-2.03-0.04) | 0.52 (-1.64-2.67) |
|  | dominant | -0.09 (-0.49-0.31) | -0.05 (-0.51-0.41) | -0.16 (-0.56-0.24) | 0.79 (-0.03-1.61) |
| rs35621 | General model a | -0.12 (-0.63-0.39) | -0.10 (-0.68-0.48) | -0.01 (-0.52-0.50) | -0.35 (-1.40-71) |
|  | General model b | 0.01 (-2.06-2.07) | 0.01 (-2.34-2.35) | 0.44 (-1.62-2.50) | 1.33 (-2.94-5.59) |
|  | dominant | -0.11 (-0.61-0.39) | -0.10 (-0.67-0.47) | 0.01 (-0.49-0.51) | -0.28 (-1.32-0.76) |

Data are presented as B (95%CI) = regression coefficient (95% confidence interval); ln=natural logarithm

The analyses are adjusted for age, gender and packyears

General model a: heterozygote versus wild type

General model b: homozygote mutant versus wild type

¶ Total cells count refers to total number of non-squamous cells in sputum

SNPs showing a significant effect are depicted in bold (p<0.05)

**Table S4b: *MRP1* SNPs and inflammatory cells in induced sputum of COPD patients**

| **SNP** | **Model** | **Ln (lymphocytes)** | **Ln (basophils)** | **Ln (epithelial cells)** |
| --- | --- | --- | --- | --- |
| rs212093 | General model a | -0.08 (-0.78-0.62) | -0.16 (-0.40-0.09) | 0.21 (-0.78-1.19) |
|  | General model b | -0.37 (-1.19-0.45) | -0.09 (-0.37-0.19) | 0.08 (-1.07-1.23) |
|  | dominant | -0.18 (-0.83-0.46) | -0.13 (-0.36-0.09) | 0.16 (-0.74-1.07) |
| rs4148382 | dominant | 0.62 (-0.07-1.32) | 0.05 (-0.19-0.29) | -0.53 (-1.50-0.45) |
| rs504348 | General model a | -0.03 (-0.71-0.65) | -0.10 (-0.33-0.14) | 0.24 (-0.71-1.19) |
|  | General model b | -0.77 (-2.88-1.33) | 0.02 (-0.72-0.75) | 1.05 (-1.90-4.01 |
|  | dominant | -0.08 (-0.74-0.58) | -0.09 (-0.32-0.14) | 0.30 (-0.62-1.22) |
| rs4781699 | General model a | -0.15 (-0.73-0.44) | -0.06 (-0.26-0.15) | -0.001 (-0.83-0.82) |
|  | General model b | -0.78 (-2.27-0.72) | -0.05 (-0.57-0.47) | 0.55 (-1.55-2.65 |
|  | dominant | -0.20 (-0.77-0.37) | -0.06 (-0.26-0.14) | 0.04 (-0.76-0.85) |
| rs35621 | General model a | -0.37 (-1.08-0.36) | 0.13 (-0.17-0.38) | -0.05 -1.07-0.96) |
|  | General model b | 1.04 (-1.87-3.94) | 0.02 (-0.99-1.04) | -0.15 (-4.26-3.96) |
|  | dominant | -0.31 (-1.02-0.40) | 0.12 (-0.13-0.37) | -0.06 (-1.05-0.94 |

Data are presented as B (95%CI) = regression coefficient (95% confidence interval); ln=natural logarithm

The analyses are adjusted for age, gender and packyears

General model a: heterozygote versus wild type

General model b: homozygote mutant versus wild type

**Table S5: *MRP1* SNPs and FEV_1_ level of COPD patients**

| **SNP** | **Model** | **FEV_1_** |
| --- | --- | --- |
| rs212093 | General model a | 0.03 (-0.12 - 0.18) |
|  | General model b | **0.22 (0.05 - 0.40)** |
|  | dominant | 0.09 (-0.05 - 0.23) |
| rs4148382 | dominant | **-0.22 (-0.36 - -0.08)** |
| rs504348 | General model a | 0.04 (-0.11 - 0.18) |
|  | General model b | 0.26 (-0.21 - 0.65) |
|  | dominant | 0.06 (-0.08 - 0.20) |
| rs4781699 | General model a | 0.06 (-0.07 - 0.19) |
|  | General model b | 0.09 (-0.17 - 0.35) |
|  | dominant | 0.07 (-0.06 - 0.19) |
| rs35621 | General model a | 0.14 (-0.02 - 0.30) |
|  | General model b | 0.09 (-0.57 - 0.75) |
|  | dominant | 0.14 (-0.02 - 0.30) |

Data are presented as B (95%CI) = regression coefficient (95% confidence interval)

FEV_1_ = forced expiratory volume in one second

The analyses are adjusted for age, gender, height and packyears

General model a: heterozygote versus wild type

General model b: homozygote mutant versus wild type

SNPs showing a significant effect are depicted in bold (p<0.05)

**Table S6: Frequencies of *MRP1* haplotypes**

| Haplotypes  (threshold > 0.01) |  | Inflammatory cells | |
| --- | --- | --- | --- |
| rs212093  rs4148382  rs504348  rs4781699  rs35621 | FEV_1_ level | bronchial biopsies | induced sputum |
| 00000 | 0.2986 | 0.3087 | 0.3099 |
| 00010 | 0.0273 | 0.0271 | 0.0291 |
| 00011 | 0.0077 | 0.0075 | 0.0078 |
| 00100 | 0.0144 | 0.0140 | 0.0075 |
| 00110 | 0.0771 | 0.0770 | 0.0751 |
| 00111 | 0.0196 | 0.0194 | 0.0213 |
| 01000 | 0.0876 | 0.0848 | 0.0917 |
| 01001 | 0.0155 | 0.0157 | 0.0166 |
| 01010 | 0.0167 | 0.0147 | - |
| 10000 | 0.2574 | 0.2555 | 0.2649 |
| 10001 | 0.0539 | 0.0530 | 0.0558 |
| 10010 | 0.0788 | 0.0756 | 0.0815 |
| 10011 | 0.0023 | 0.0031 | 0.0027 |
| 10101 | 0.0021 | 0.0022 | 0.0036 |
| 10110 | 0.0412 | 0.0417 | 0.0325 |

0 = wild type of the corresponding SNP; 1= minor allele carriers of the corresponding SNP

**FIGURE**

**Figure S1: Linkage disequilibrium plot and correlation coefficients (r^2^) for 5 *MRP1* polymorphisms genotyped in COPD patients (n=110)**

*MRP1 = multidrug resistance-associated protein-1* (official name *ABCC1, ATP-binding cassette (ABC) subfamily C, member 1*)

The location of the single nucleotide polymorphisms is given for the HapMap Data Release February 2, 2009

**Reference list**

S1. Siedlinski M, Postma DS, van Diemen CC, Blokstra A, Smit HA, Boezen HM:

**Lung function loss, smoking, vitamin C intake, and polymorphisms of the glutamate-cysteine ligase genes.** *Am J Respir Crit Care Med* 2008, **178:**13-19.

S2. Lapperre TS, Postma DS, Gosman MM, Snoeck-Stroband JB, ten Hacken NH, Hiemstra PS, Timens W, Sterk PJ, Mauad T: **Relation between duration of smoking cessation and bronchial inflammation in COPD.** *Thorax* 2006, **61:**115-121.

S3. Willemse BW, ten Hacken NH, Rutgers B, Postma DS, Timens W: **Association of current smoking with airway inflammation in chronic obstructive pulmonary disease and asymptomatic smokers.** *Respir Res* 2005, **6:**38.
