# Supplementary figures and images for: Multidrug resistance-associated protein-1 (MRP1) genetic variants, MRP1 protein levels and severity of COPD
Source: Respir Res. 2010 May 20;11(1):60. doi: 10.1186/1465-9921-11-60 (PMC2882908; doi:10.1186/1465-9921-11-60)

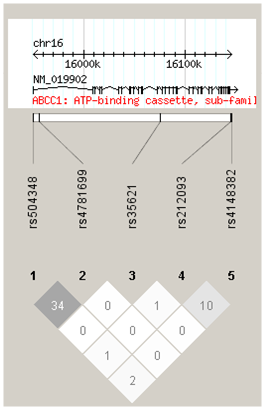

Supplement: Additional file 2 — Figure S1: Linkage disequilibrium plot and correlation coefficients (r2) for 5 MRP1 polymorphisms genotyped in COPD patients (n = 110). [file 1465-9921-11-60-S2.PNG]
